# *Tradescantia* response to air and soil pollution, stamen hair cells dataset and ANN color classification

**DOI:** 10.3389/fdata.2024.1384240

**Published:** 2024-05-15

**Authors:** Leatrice Talita Rodrigues, Barbara Sanches Antunes Goeldner, Emílio Graciliano Ferreira Mercuri, Steffen Manfred Noe

**Affiliations:** ^1^Graduate Program of Environmental Engineering, Federal University of Paraná, Curitiba, Brazil; ^2^Department of Environmental Engineering, Federal University of Paraná, Curitiba, Brazil; ^3^Institute of Forestry and Engineering, Estonian University of Life Sciences, Tartu, Estonia

**Keywords:** ResNet, VGG, air, soil, water pollution, biomonitor

## Abstract

*Tradescantia* plant is a complex system that is sensible to environmental factors such as water supply, pH, temperature, light, radiation, impurities, and nutrient availability. It can be used as a biomonitor for environmental changes; however, the bioassays are time-consuming and have a strong human interference factor that might change the result depending on who is performing the analysis. We have developed computer vision models to study color variations from *Tradescantia* clone 4430 plant stamen hair cells, which can be stressed due to air pollution and soil contamination. The study introduces a novel dataset, Trad-204, comprising single-cell images from *Tradescantia* clone 4430, captured during the *Tradescantia* stamen-hair mutation bioassay (Trad-SHM). The dataset contain images from two experiments, one focusing on air pollution by particulate matter and another based on soil contaminated by diesel oil. Both experiments were carried out in Curitiba, Brazil, between 2020 and 2023. The images represent single cells with different shapes, sizes, and colors, reflecting the plant's responses to environmental stressors. An automatic classification task was developed to distinguishing between blue and pink cells, and the study explores both a baseline model and three artificial neural network (ANN) architectures, namely, TinyVGG, VGG-16, and ResNet34. *Tradescantia* revealed sensibility to both air particulate matter concentration and diesel oil in soil. The results indicate that Residual Network architecture outperforms the other models in terms of accuracy on both training and testing sets. The dataset and findings contribute to the understanding of plant cell responses to environmental stress and provide valuable resources for further research in automated image analysis of plant cells. Discussion highlights the impact of turgor pressure on cell shape and the potential implications for plant physiology. The comparison between ANN architectures aligns with previous research, emphasizing the superior performance of ResNet models in image classification tasks. Artificial intelligence identification of pink cells improves the counting accuracy, thus avoiding human errors due to different color perceptions, fatigue, or inattention, in addition to facilitating and speeding up the analysis process. Overall, the study offers insights into plant cell dynamics and provides a foundation for future investigations like cells morphology change. This research corroborates that biomonitoring should be considered as an important tool for political actions, being a relevant issue in risk assessment and the development of new public policies relating to the environment.

## 1 Introduction

Biomonitoring is a method to complement air quality monitoring networks or chemical analysis of soils and the data provided by bioindicators can help assess the level of pollution in the environment and possibly its source (Kienzl et al., 2003). Physicochemical measurements are straightforward and precise; however, bioindicators provide assessment for interrelated effects on the environment which can help to formulate policies and regulations for the protection of human life, flora, and fauna (Mulgrew and Williams, 2000). Bioindicators are becoming more important for environmental control and impact assessments, providing information of great political relevance and making it possible to measure if intervention actions are having the desired results (Kienzl et al., 2003; Cozea et al., 2019).

Plant cells can change color due to environmental stress factors such as pH, temperature, light intensity, and nutrient availability (Chapin et al., 1987; Młodzińska et al., 2009; Hu, 2013), which affect the presence of pigments such as chlorophyll (Vogelmann and Evans, 2002), carotenoids (Langi et al., 2018), and anthocyanins (Nassour et al., 2020). *Tradescantia pallida* via Trad-MCN bioassay (Carreras et al., 2006; Prajapati and Tripathi, 2008) and *Tradescantia* clone 4430 via Trad-SHM bioassay (Rodrigues et al., 2023) revealed sensibility to air quality, and these studies show that plants exposed to sites with highest traffic volumes had higher frequencies of micronuclei and color change in stamen hair compared with the control area. *Tradescantia* demonstrated genotoxicity of ambient air due to ionizing radiations (Ichikawa et al., 1969; Ichikawa, 1992; Panek et al., 2011), Caldwell et al. (1974) performed field and laboratory experiments showing response of *Tradescantia* plants to elevated intensities of global UV-B radiation. An increased frequency of micronuclei (Trad-MCN) was detected in *Tradescantia* clone 4430 planted on soils contaminated with metals or fly ash from coal-fired power stations (Čėsnienė et al., 2017; Meravi and Prajapati, 2018), and soil contaminated by diesel also showed influence on stamen hair cells of the plant (Green et al., 1996; Goeldner, 2023). Khosrovyan et al. (2022) used *Tradescantia* clone 02 and the Trad-SHM and Trad-MCN bioassays to check the water quality of an urban river that runs through a highly urbanized and industrial area and observed an increase in all the parameters studied, as well as morphological changes such as an increase in pink cells and tetrads with micronuclei compared with the negative control (tap water).

Several authors developed algorithms for human and vegetal cell segmentation from images such as Contour Proposal Networks (CPNs) (Upschulte et al., 2023), U-Net, and DeepCell (Caicedo et al., 2019). Although there are a lot of literature studies for general image classification (Wieslander et al., 2017; Ikechukwu et al., 2021; Jusman, 2023), we have found few articles for the classification of cells, such as malaria-infected cells (Loddo et al., 2019; Reddy and Juliet, 2019), and no research use computer methods for classifying colors of *Tradescantia* plant cells. In addition, no microscopy dataset of *Tradescantia* single-cell images was found on research databases.

Stamen hair cell color anomalies counting serves as a proxy for analyzing whether the plant has been exposed to any stressors. Trad-SHM bioassay is traditionally done through manual counting and is a time-consuming process involving collecting the material, mounting the slide, observing it under the microscope, counting the cells, and then analyzing the data. To improve this process and reduce the examiner's workload and increase the assertiveness of cell counting process, image processing with color recognition was thought to optimize the manual counting process. However, computational classification of *Tradescantia* cells as blue or pink is not a trivial task. Visually, human eyes can easily distinguish between the two colors on the macro scale of the cell, but when observing the pixels closely, it is clear that there are many blue pixels in a pink cell and there are many pink pixels in a blue cell. Furthermore, there is great variability in the blue and pink tones from cell microscopy images. This study has three principal aims: (i) to investigate the sensibility of *Tradescantia* to air pollution and soil contamination; (ii) to build a novel dataset composed of single cell images from *Tradescantia* clone 4430 plant, and (iii) to apply neural network architectures (TinyVGG, VGG-16, and ResNET34) capable of automatically classify cells into blue or pink classes. The idea is to build artificial intelligence algorithms that might allow fast identification of blue and pink cells in stamen hair images of *Tradescantia* plants and facilitate quantification for subsequent statistical analysis. From an environmental point of view, by reducing time in the laboratory, there is the possibility of increasing the sampling area, covering possibly unseen critical areas via biomonitoring air, water, and/or soil quality. Furthermore, our dataset can be used as a resource for testing and validating automated image-analysis algorithms.

## 2 Materials and methods

### 2.1 Experiments and *Tradescantia* single cell dataset

**Figure 1:**
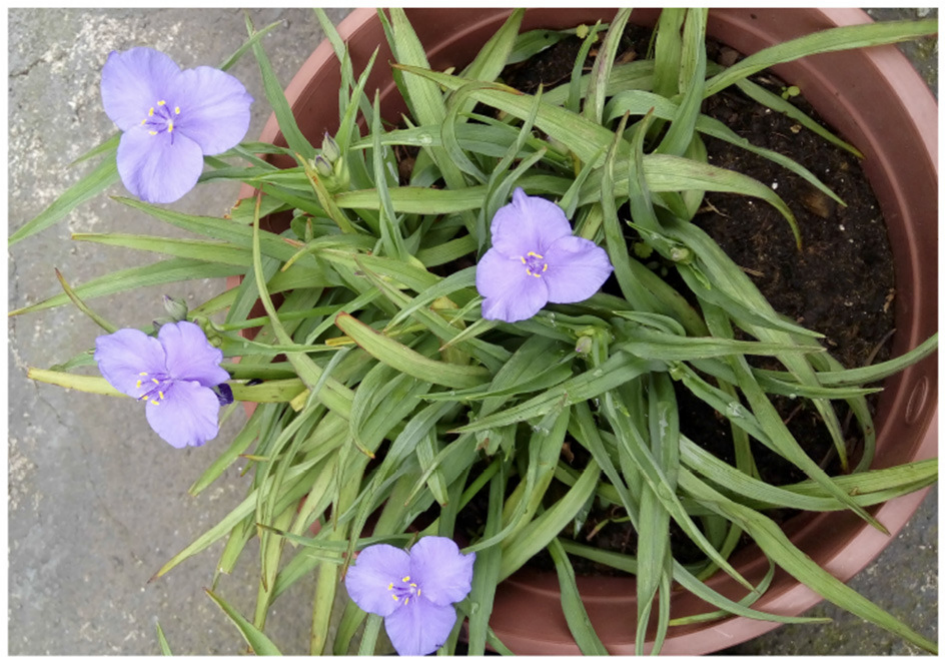
*Tradescantia* clone 4430 plant from the experiment on air pollution and particulate matter in Curitiba, Brazil (Rodrigues et al., 2023).

Here, we describe the experiments that give birth to the Trad-204 dataset, a computer vision single cell *Tradescantia* clone 4430 set of images. The pictures represent stamen hair cells of *Tradescantia* clone 4430 plant that were captured during the Trad-SHM bioassay. This assay is indicated to identify changes in cell color from blue to pink, which indicates that the plant was exposed to some types of environmental stress, such as temperature or pH change, radiation, and contamination of soil, water, or air (Underbrink et al., 1973; Sparrow et al., 1974; Schairer et al., 1978, 1982; Ma et al., 1994). This change in color can be a result of mutation (Meravi and Prajapati, 2018) and/or can be associated with anthocyanin pigments that are responsible for red, purple, and blue colors, and they act as antioxidants and may play a role in protecting plants from damage caused by UV light, pathogens, and herbivores (Nassour et al., 2020). Figure 1 shows an exemplar of the *Tradescantia* clone 4430 plant.

The experiments that generated the images were related to the biomonitoring of air quality in Curitiba and Araucária-PR, Brazil, from 2020 to 2021 (Rodrigues et al., 2023), and the biomonitoring of soil quality after contamination by diesel oil in different concentrations, and this last experiment was carried out in the Federal University of Paraná (UFPR) laboratory from 2022 to 2023 (Goeldner, 2023). Rodrigues et al. (2023) deployed eight monitoring points and a control to compare *Tradescantia* clone 4430 bio-monitoring with particulate matter measurements using the SDS011 optical sensor. Each monitoring point contained five pots of *Tradescantia* plants that were acclimatized for 2 months. The control box was sealed at the top with filter paper to isolate and prevent particles from coming into contact with the plants. Throughout the monitoring period, each pot was watered weekly with approximately 100 ml of water. After the acclimatization period, inflorescences were collected for stamen hair analysis using the Trad-SHM bioassay technique. Goeldner (2023) prepared five pots with soil and stems of *Tradescantia* clone 4430. Concentrations of diesel oil per kilo of soil were: zero (control), 100, 1.000, 10.000, and 100.000 mg/kg. After this contamination, the pots were placed on a bench in a laboratory. After a period of 3 weeks, the flowers began to be collected for analysis of the stamen hair using the Trad-SHM bioassay.

To quantify the dose-response relation of diesel oil in *Tradescantia*, we propose a saturating function or stimulus response curve, which is described in Equation (1). The function increases at first but only up to a maximum (saturation) level.


(1) $$\begin{array}{l} y=m\cdot \frac{x}{h+x}+b \end{array}$$

In Equation (1), *m* is the saturation level, or the value that *y* approaches as *x* gets large; the constant *h* is the half-saturation point, the *x* value at which $y=\frac{m}{2}$; and the last parameter *b* is a bias or error, which is supposed to be very small (Crump et al., 1976).

**Figure 2:**
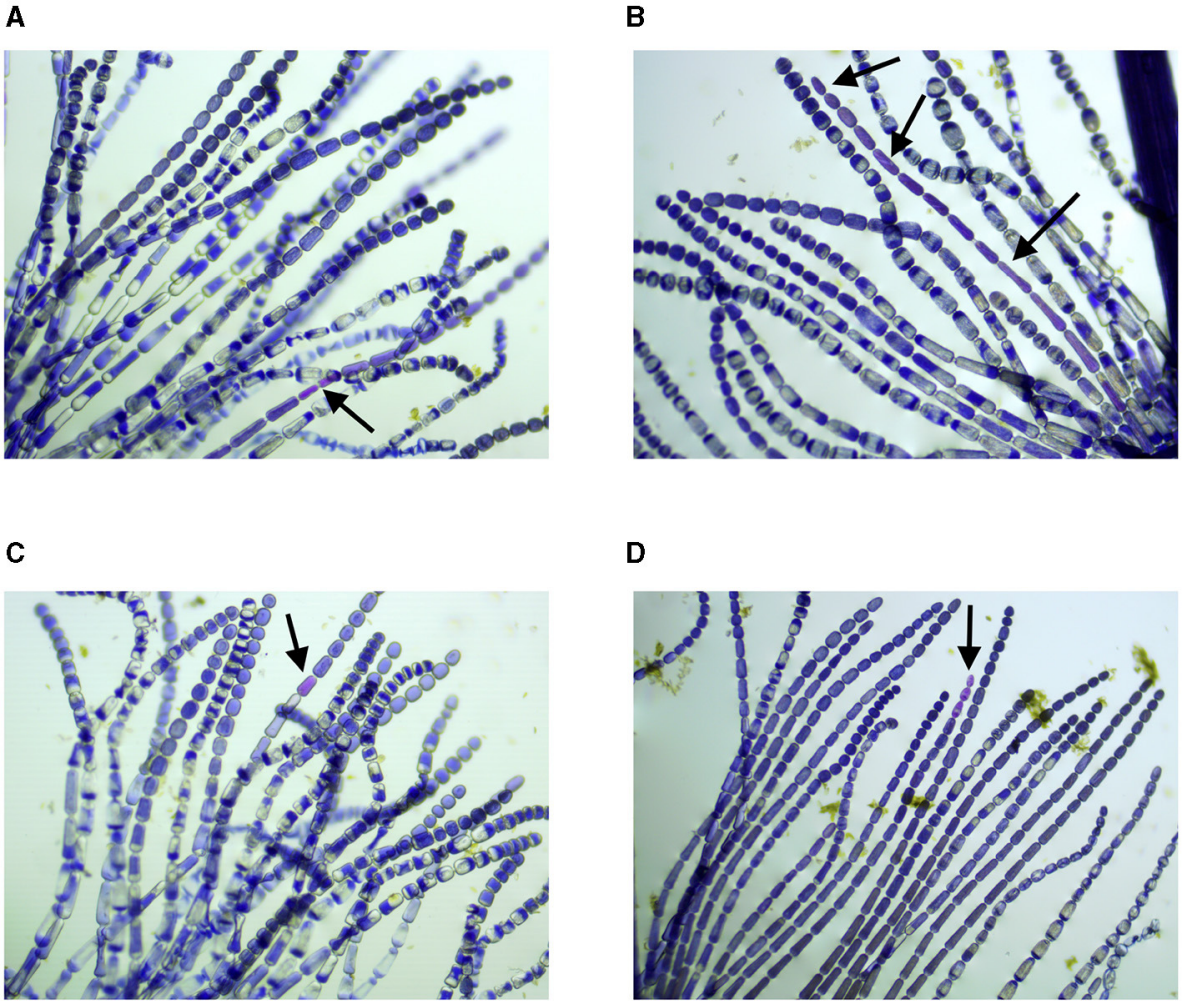
Stamen hair photographs, samples were collected on: **(A)** 02/12/2022, **(B)** 29/11/2022, **(C)** 08/12/2022, and **(D)** 05/01/2023. Black arrows indicate cells with modification of color (Goeldner, 2023). **(A)** Sample collected on 02/12/2022, control plant specimen planted on soil free of diesel contamination. **(B)** Sample collected on 29/11/2022, plant specimen planted on soil contaminated with 1,000 mg of diesel. **(C)** Sample collected on 08/12/2022, plant specimen planted on soil contaminated with 100 mg of diesel. **(D)** Sample collected on 05/01/2023, plant specimen planted on soil contaminated with 100 mg of diesel.

The preparation procedures for the Trad-SHM bioassay (Goeldner, 2023; Rodrigues et al., 2023) followed those described in the study by Underbrink et al. (1973) with some adaptations. The flowers were always collected in the morning, as they close up and wilt in the afternoon, and placed in pots labeled with their origin. Afterward, all the stamens were removed with the help of tweezers and arranged on slides, which were then identified according to where the flowers had been collected. A 1:1 solution of 70% alcohol and glycerin was used to fix the stamens. With the aid of tweezers and a needle and observed with a magnifying glass, the stamen hairs were aligned on the slide. After this procedure, the material was analyzed using an optical microscope to obtain images of the stamen hairs. Photographs of cells photographs were taken using a camera attached to a binocular optical microscope. To create the database, entitled Trad-204, images that contained pink and blue cells in the same frame were used. In total, 31 multiple cell images were used, including 16 pictures from the air quality study (Rodrigues et al., 2023) and 15 images from the contaminated soil research (Goeldner, 2023). Figure 2 shows four pictures from the study by Goeldner (2023), and images were captured using ToupView software.

The Trad-204 can be used to study the color and format anomalies of *Tradescantia* cells. The dataset is a labeled set of 204 single cell images, 106 from soil contamination study and 98 from air quality research, and all cell pictures were manually cropped using Gimp (GNU Image Manipulation Program) by the authors. Trad-204 dataset consists of color images labeled in two classes, blue and pink cells, with 102 images per class. There are 164 training and 40 test images. Image shapes range from 13 to 256 pixels in height or weight.

Data preprocessing steps involved images normalization and resizing of images, and no augmentation was performed. The individual cell pictures were transformed and organized in batches to serve as inputs to each ANN model training and testing. For TinyVGG, the images were resized to 64 × 64 pixels with 3 RGB color channels; for ResNET34, the images were resized to 224 × 224 pixels and normalized using the RGB mean of (0.4914, 0.4822, and 0.4465) and standard deviation of (0.2023, 0.1994, and 0.201); for VGG16, the images were resized to 227 × 227 pixels with 3 RGB color channels. The image resize values were distinct due to the different architecture of each ANN model.

### 2.2 Models for color classification

**Figure 3:**
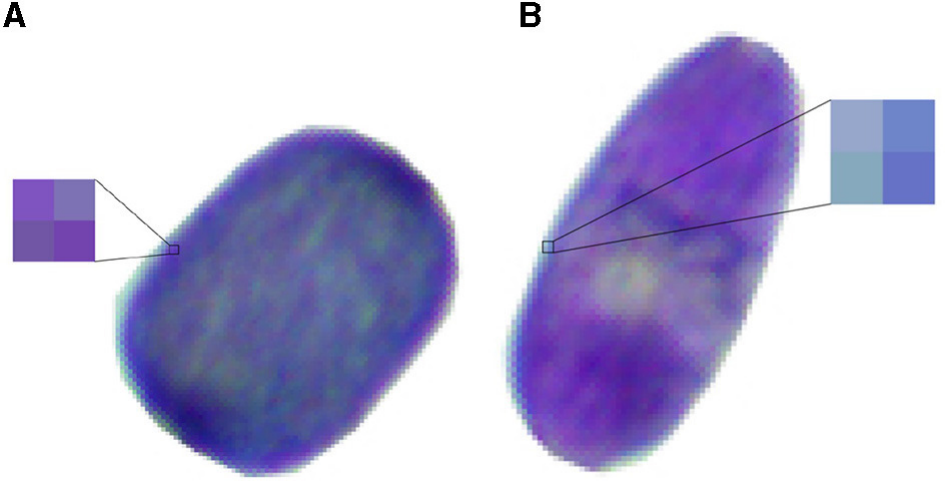
Sample of blue and pink cells **(A)** Blue cell with pinkish pixels. **(B)** Pink cell with blueish pixels.

As mentioned before, there is a great variety of colors and tonalities in the image dataset. In addition, there are cells with different formats, ranging from round and oval to squared shapes. One key aspect for the color classification is that there is blue cell with pinkish pixels and a pink cell with blueish pixels, as shown in Figure 3.

For the color classification task, we have used part of the Trad-204 dataset containing 106 images of individual cells and the subset of cell pictures from soil contamination study. This set was used to compare the baseline model and the different neural network architectures. A random split was created with 84 single cell images for training 22 single cell images for testing.

#### 2.2.1 Baseline model

A simple baseline model based on pixel color channels was developed to classify the *Tradescantia* cells into pink or blue cells. We used the Red, Green, and Blue (RGB) color channels to define mean values for blue and pink cells. The mean $\bar{X}$ and standard deviation σ of each color was calculated based on samples from 10 random images of the dataset. A pink pixel in the RGB system was calculated as (133.83 ± 27, 75.84 ± 27, and 182.24 ± 27), where each color was represented as mean ± standard deviation. A blue pixel was determined as (70.99 ± 27,75.87 ± 27, and 163.05 ± 27). If the pixel RGB colors falls into these intervals, it is counted either as blue or pink. In the end, we performed the sum of blue and pink pixels, and the highest number defines the major color of the cell.

#### 2.2.2 Neural network architectures

**Figure 4:**
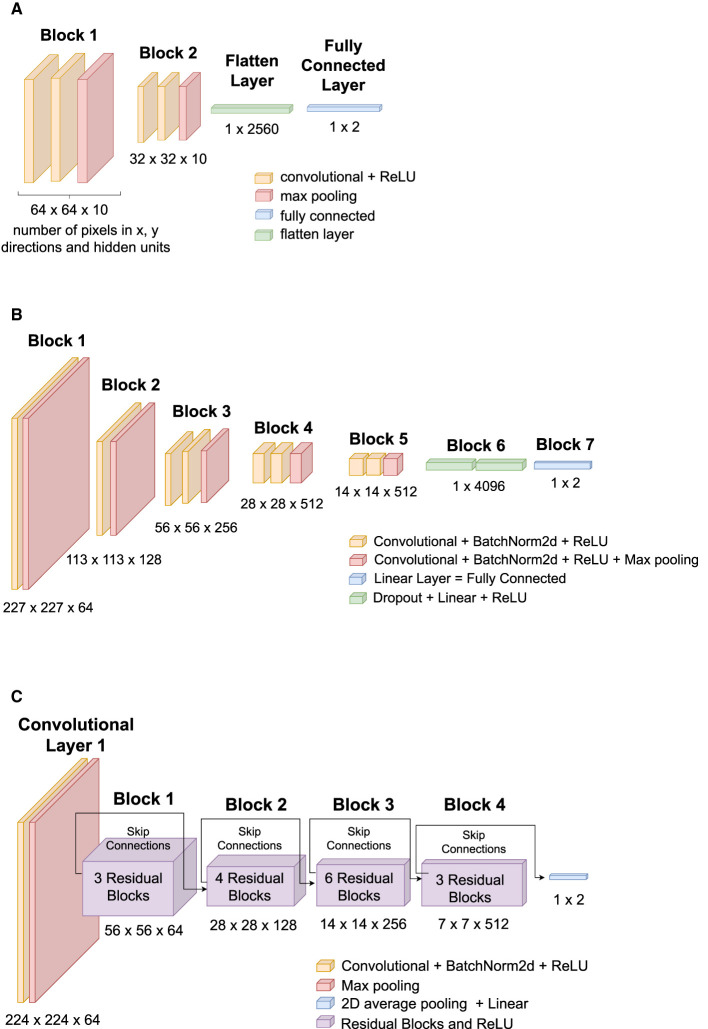
ANNs architectures used in this study: **(A)** TinyVGG, **(B)** VGG16, and **(C)** ResNet34. **(A)** TinyVGG architecture composed by convolutional, rectified linear units (ReLU), max pooling, fully connected, and flatten layers. **(B)** VGG16 architecture composed by convolutional, batch normalization (BatchNorm2d), rectified linear units (ReLU), max pooling, fully connected, and dropout layers. **(C)** ResNet34 architecture composed by convolutional, batch normalization (BatchNorm2d), rectified linear units (ReLU), max pooling, 2d average pooling, fully connected, and residual layers.

Three Artificial Neural Network (ANN) architectures were compared: TinyVGG (8 layers), VGG16 (16 layers), and ResNet34 (34 layers). VGG stands for Visual Geometry Group, and ResNET is an abbreviation of Residual Networks. A Residual Network is an ANN with skip connections that perform identity mappings, which are merged with the layer outputs by the addition of the study by He et al. (2016). Figure 4 shows the architectures of each ANN used in our research, and the diagrams show how a 2 dimensional image is processed by convolutions to be classified into two classes (blue or pink). The numbers below each block in Figures 4A–C describe the image size in pixels and the number of hidden units to produce a classifier, for example, the first block of the TinyVGG accepts images of 64 × 64 pixels and have 10 neurons in the hidden layer. Labels with different colors indicate which kind of layer each architecture of ANN is using. All ANN models were programmed using *PyTorch* library and executed in CUDA (or Compute Unified Device Architecture) graphics processing units (GPUs). The 3 ANN architectures were trained for 20 epochs and we did not used data augmentation.

Our TinyVGG, as shown in Figure 4A, has two blocks of a convolutional layer and a rectified linear unit (ReLU) activation function followed by a Flatten layer and a Linear layer. We have used a batch size of 32 images to train the model. The convolutional layers apply 2D convolutions over the input signal with kernel size of 3, stride equals of 1, and padding of 1. The MaxPool2d layer has hyper parameters: kernel size = 2 and stride = 2. The loss function or criterion was selected as the cross entropy loss between input logits and target. The optimizer was set to Adam algorithm (Kingma and Ba, 2014) with learning rate 0.001.

The VGG is based on the study of AlexNet (Krizhevsky et al., 2012), and it focuses on depth of Convolutional Neural Networks (CNNs) (Simonyan and Zisserman, 2014). Our architecture, as shown in Figure 4B, consists of 16 convolutional layers (VGG-16) and its convolutional layers have 3x3 filters. We have used a batch size of 32 images to train the model. It has five blocks of convolutional layers followed by Batch Normalization and a ReLU activation function. Then, it follows with a Dropout layer, a Linear layer, and a ReLU activation function. The criterion was chosen as the cross entropy loss, and the optimizer is stochastic gradient descent (Sutskever et al., 2013) with learning rate of 0.005.

ResNet has VGG's full 3 × 3 convolutional layer design. The residual block has two 3 × 3 convolutional layers with the same number of output channels. Each convolutional layer is followed by a batch normalization layer and a ReLU activation function. Then, we skip these two convolution operations and add the input directly before the final ReLU activation function (Zhang et al., 2023).

The first step on the ResNet consists on a convolution, batch normalization, and max pooling operation. Then, the core building blocks of ResNet are residual blocks. ResNet34 is composed of multiple residual blocks stacked together. Each residual block consists of two convolutional layers, batch normalization, and a shortcut connection (skip connection). The skip connection allows the gradient to bypass the convolutional layers, mitigating the vanishing gradient problem. The identity mapping helps in learning residual functions, making it easier to train deeper networks (Zhang et al., 2023).

Figure 4C shows the architecture for our Residual Network, ResNet34. A batch size of 32 images was used to train the model. Criterion was chosen as cross entropy loss, and the optimizer is stochastic gradient descent (Sutskever et al., 2013) with learning rate of 0.01.

#### 2.2.3 Evaluation metrics

Loss function cross entropy loss was used to evaluate both training and testing of the ANNs. Another metric used to evaluate all models was Accuracy, which is defined by the Equation (2):


(2) $$\begin{array}{l} \text{Accuracy}=\frac{\left(\text{TP}+\text{TN}\right)}{\left(\text{P}+\text{N}\right)} \end{array}$$

The terminologies of Equation (2) are True Positive (TP)—the model predicted “pink” and its actual class is “pink”; False Positive (FP)—the model predicted “pink” and its actual class is “blue”; False Negative (FN)—the model predicted “blue” and its actual class is “pink”; True Negative (TN)—the model predicted “blue” and its actual class is “blue”. These are the performance criteria calculated from the confusion matrix. The remaining symbols are: P = TP+FN and N = TN+FP.

## 3 Results

This section is divided into three parts: First, we show the sensibility of *Tradescantia* to air pollution and diesel oil soil contamination, and then, we describe the single cell dataset developed in this study and, in the last part, neural network architectures for color classification.

### 3.1 *Tradescantia* sensibility to air and soil stress factors

**Figure 5:**
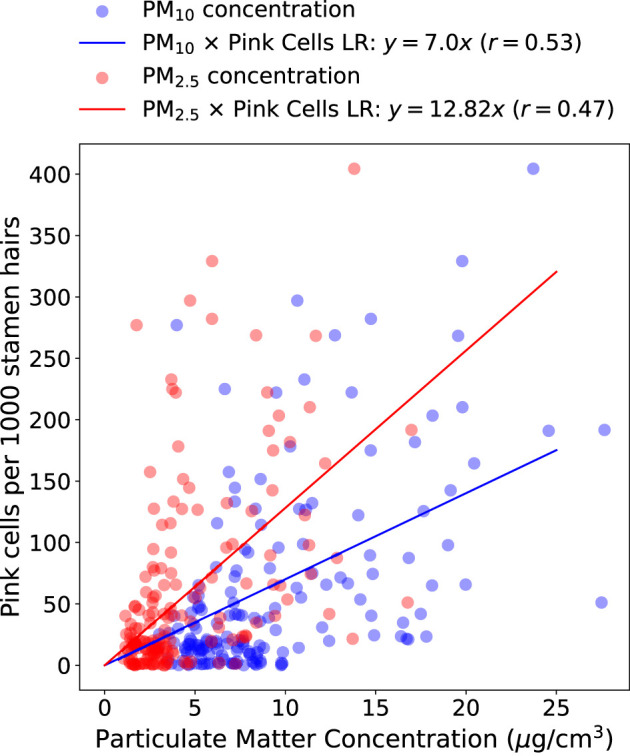
Average particulate matter concentration (PM_10_ and PM_2.5_) calculated with BSW method and pink cell appearance per 1000 stamen hairs of *Tradescantia* clone 4430. Linear Regression (LR) equations and Pearson's correlation coefficients (*r*) are represented above the graph.

Figure 5 represents the results from the study by Rodrigues et al. (2023) with data from four sampling points (Jardim Botânico, Jardim das Américas, Mercês, and Orleans) measured between 2020 and 2021 in Curitiba, Brazil. It shows a scatter plot of particulate matter (PM_2.5_ and PM_10_) average concentrations and a measure of pink cells appearance obtained from Trad-SHM bioassay. Backward Sliding Window (BSW) Method (Rodrigues et al., 2023) was used for detecting a 6-day exposure window and 2-day lag time before inflorescence sampling, and this method was used to calculate the averages of PM_2.5_ and PM_10_ as proxy for air pollution exposure. The graph also shows linear regression between particulate matter (PM) and pink cell appearance per 1000 stamen hairs. For PM_10_ and PM_2.5_, the equations *y* = 7.00*x* and *y* = 12.82*x*, respectively, represent the pink cell appearance as the dependent variable *y* and average PM concentrations as independent variable *x*. Pearson correlation coefficient was calculated demonstrating positive correlation between the air pollution proxy and the change of color in cells.

The test chosen to evaluate the reaction of *Tradescantia* clone 4430 plants to different concentrations of diesel was the Trad-SHM bioassay (Sparrow et al., 1971; Maziviero, 2011). The inflorescences of *Tradescantia* clone 4430 began to be collected and analyzed on 3 November 2022, 3 weeks after the start of the experiment. On 31 October 2022, the plant in the pot with a concentration of 100,000 mg/kg began to wilt and show yellowish leaves and stems, on 17 November 2022, the plant was completely wilted, predominantly brown in color with slightly oily leaves, and on 3 December 2022, the plant was already dry. For the 100,000 mg/kg concentration, it was not possible to collect any inflorescences.

**Figure 6:**
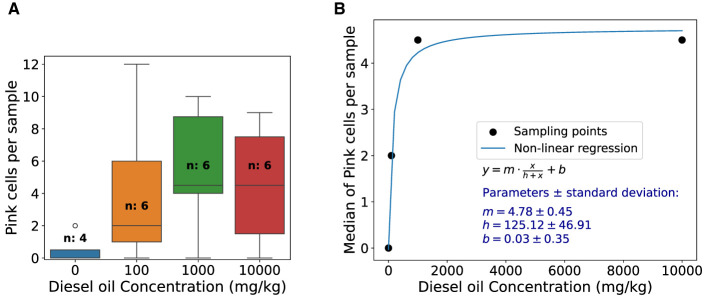
Pink cell appearance box-plot and dose-response function of *Tradescantia* exposure to diesel oil-contaminated soil. **(A)** Box plot of pink cells appearance in each treatment of diesel oil-contaminated soils. The control is represented by the zero oil concentration and “n” represents the number of stamen hair samples analyzed. **(B)** Median of pink cells appearance per sample and dose response functional behavior of *Tradescantia* exposure to diesel oil-contaminated soil. Function used for fitting, parameters, and standard deviation errors is shown in the graph.

The results of soil contamination study are presented in Figure 6. A box plot of pink cell appearance in each treatment of diesel oil-contaminated soils is presented in Figure 6A, showing the data dispersion. Figure 6B reveals the median of pink cell appearance per sample and dose response functional behavior of *Tradescantia* exposure to diesel oil-contaminated soil. After fitting Equation (1) to the data, we found *y* = 4.78*x*/(125.12+*x*)+0.03, and parameters and standard deviation errors are displayed in the graph.

### 3.2 Trad-204 dataset

**Figure 7:**
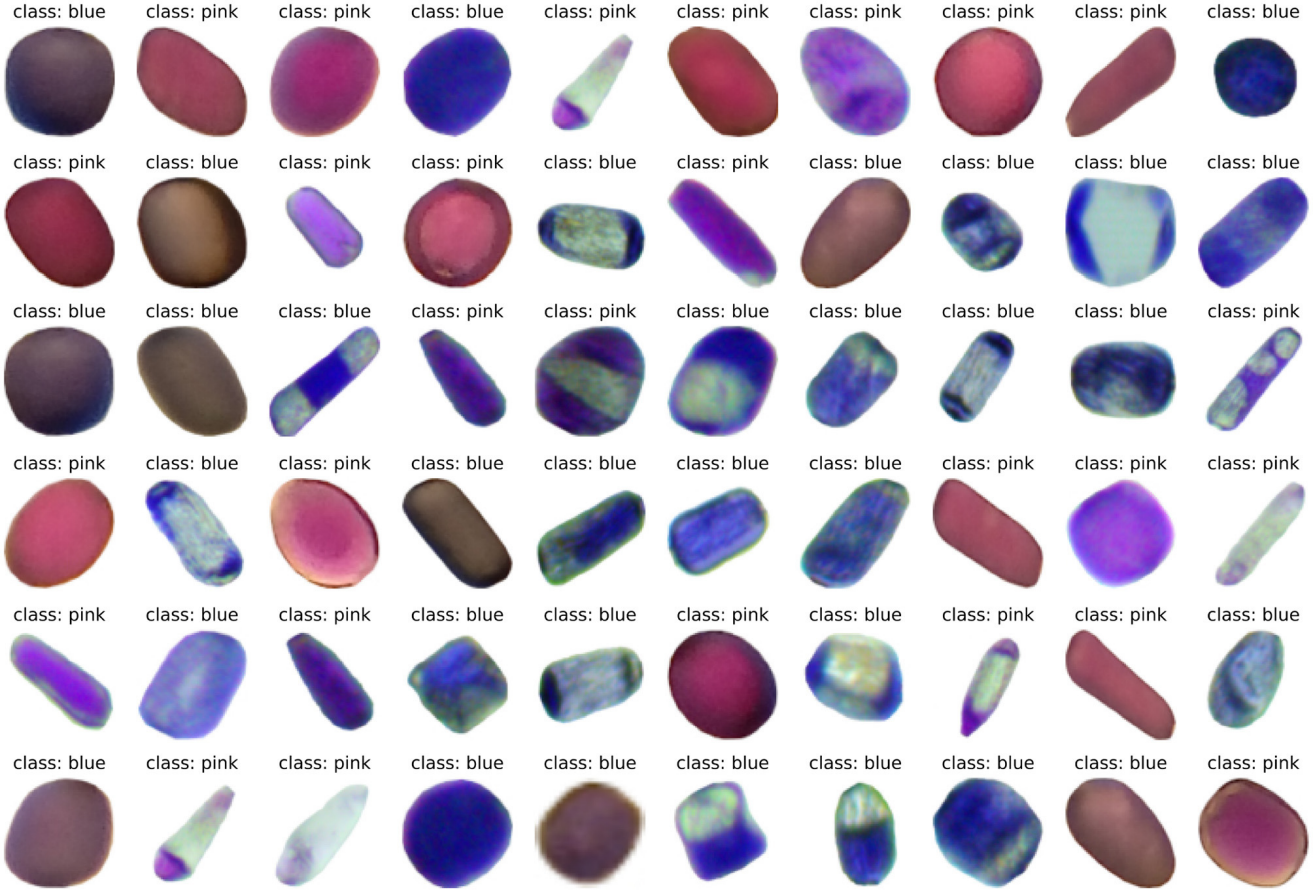
Trad-204 random sample of 60 cells with labels.

In this section, we show some images of the constructed dataset and the results from baseline and ANN color classification task. Figure 7 shows a random sample of 60 cells from the Trad-204 dataset.

**Figure 8:**
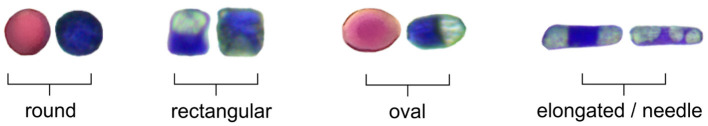
Different cells format: round, rectangular, oval, and elongated or needle types.

Figure 8 shows format of different cells from the dataset. The shapes vary between round, rectangular, oval, and elongated/needle types. There is normally a progression of decreasing cell size and shape from large basal cells to very small cells in the more distal positions of the hair and a decrease in cell size from the base of the filament to the anther (Underbrink et al., 1973). In addition, some cells show vacuoles inside of the cytoplasm, which might have some effect on the pattern recognition from ANN.

Table 1 shows descriptive statistics from Trad-204 dataset. It describes information from pixels that contain cells, excluding white pixels around cells.

**Table 1:** Trad-204 dataset descriptive statistics.

**Statistics**	**Blue cells**	**Pink cells**
Number of images	102	102
Max number of pixels per image	19,875	26,106
Min number of pixels per image	381	290
Average number of pixels per image	8,353.6	6,671.8
Per channel	(R, G, B)	(R, G, B)
Average maximum pixel value	(154.8, 178.1, 207.5)	(221.0, 208.7, 222.4)
Average minimum pixel value	(37.9, 32.1, 28.8)	(84.7, 25.3, 66.3)
Average pixel value	(97.6, 91.7, 122.0)	(143.9, 97.1, 140.2)
Standard Deviation	(25.0, 31.8, 45.7)	(33.6, 34.1, 39.4)

### 3.3 ANN color classification

**Figure 9:**
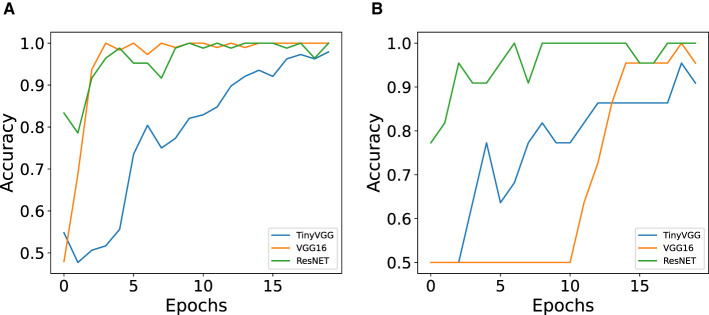
Comparison of train and test accuracy between ANN models (TinyVGG, VGG16, and ResNET) for color classification. **(A)** Train accuracy for the three ANN architectures. **(B)** Test accuracy for the three ANN architectures.

From this part onward, we present the results for classifying cell colors from the dataset with 106 images. Figure 9 shows a comparison of train and test accuracy between ANN models. It can be observed that during training, TinyVGG takes more epochs to achieve high accuracy in comparison with VGG16 and ResNET. Training accuracy shows how efficient the network is at correctly classifying the data it is being trained on (Theckedath and Sedamkar, 2020). VGG16 shows instability in the test, and ResNET has the better performance, both in training and testing. Accuracy validation is the most important because it indicates the network's success in correctly classifying data that had not been classified before (Theckedath and Sedamkar, 2020).

The performance of baseline model and each neural network architecture is presented in the Table 2. All ANN models were trained with 20 epochs, and we decided to use and show the first 20 epochs because it was the optimization period that took the three architectures to reach at least 90% of accuracy. The VGG16 and ResNET34 architectures achieved 100% accuracy in less than 10 training epochs. The total training time and the number of epochs to achieve the best accuracy varied according to the structure of each model. For TinyVGG, the total training time was approximately 9 s, for VGG16 the total time was approximately 47 s, and for ResNET34, this time was approximately 36 s. Although TinyVGG took less time to train, this model used more epochs to achieve greater accuracy in correctly classifying the training data. VGG16 took longer to train, but its accuracy results are intermediate compared with the other two ANN models. ResNET34, which showed the best performance in training and testing, had an intermediate training time between the other two ANN models, as shown in Table 2 and Figures 9A, B.

**Table 2:** Models training time and accuracy for train and test sets.

**Model**	**Train accuracy**	**Test accuracy**	**Training time (seconds)**
Baseline	0.67	0.87	-
TinyVGG	0.98	0.91	9
VGG-16	1.00	0.95	47
ResNET	1.00	1.00	36

## 4 Discussion

Bioindicators are organisms that are used to assess the environmental quality of a site, as well as the impact that a given pollutant has on the ecosystem. They are usually applied to specific sites, with local sources of pollutants, and can provide information for implementing actions to reduce pollutant emissions. To be relevant for political and administrative decisions, bioindicators must provide simple, easy-to-interpret information about the environment in which they are inserted, responding to environmental changes that have occurred as a result of anthropogenic activity and showing a relevant integration between economic and environmental issues (Kienzl et al., 2003).

*Tradescantia* revealed positive correlation for PM_2.5_ and PM_10_ and a saturation dose-response behavior for diesel-contaminated soil, as shown in Figures 5, 6. This result is aligned with other studies (Green et al., 1996; Carreras et al., 2006; Prajapati and Tripathi, 2008). The Pearson correlation was higher between pink cell appearance and PM_10_ when compared with PM_2.5_, which indicates that *Tradescantia* may be more sensitive to coarse particles than finer ones. Guimarães et al. (2004) also found a positive correlation (r = 0.47) between PM_10_ and changes in the pink color of the stamen hair of *Tradescantia* clone KU-20, as did Ferreira et al. (2003), who found a positive correlation (r=0.41) between particulate matter and the frequency of color changes in stamen hair in a study with *Tradescantia*. An analysis of the particle composition would be crucial to identify the substances present in PM_2.5_ and PM_10_, and this is certainly a limitation of this study.

Figures 7, 8 show different cell colors, formats, and patterns, and it is the first single cell image dataset for *Tradescantia* clone 4430. The architectures of ANNs tested here were capable of reading, learning, and generalizing the classification of cell colors with vacuoles of different sizes. Vacuoles are multifunctional organelles of plant cells, which can vary largely in size depending on the amount of available water. They are lytic compartments, function as reservoirs for ions and metabolites, such as pigments, and are crucial to processes of detoxification and general cell homeostasis (Zhang et al., 2014; Kaiser and Scheuring, 2020). They are involved in cellular responses to environmental and biotic factors that provoke stress (Marty, 1999).

The vacuole plays a role to maintain pressure against the inside of cell wall, giving the cell shape and helping in support. Turgor pressure within cells is regulated by osmosis, and this also causes the cell wall to expand. Along with size, rigidity of the cell is also caused by turgor pressure; a lower pressure results in a wilted cell (Fricke, 2017). The plant's turgor pressure is regulated by the cell's semipermeable membrane, selectively permitting certain solutes to enter and exit the cell, thereby sustaining a minimum pressure (Steudle et al., 1977). Other mechanisms include transpiration, which results in water loss and decreases turgidity in cells (Waggoner and Zelitch, 1965). Turgor pressure is also a large factor for nutrient transport throughout the plant. Different cell formats found on the Trad-204 dataset (Figure 8) might be related to turgor pressure inside cells.

Turgidity occurs when the membrane of the cell exerts pressure against the cell wall, resulting in high turgor pressure, or more rounded cells. Conversely, low turgor pressure leads to cell flaccidity and rectangular shape, which is evident in plants through wilted anatomical structures–a phenomenon known as plasmolysis (Stadelmann, 1966). The volume and geometry of the cell influence turgor pressure, impacting the plasticity of cell wall. Research indicates that smaller cells undergo a more pronounced elastic change compared with larger cells (Steudle et al., 1977). Turgor pressure also plays a crucial role in plant cell growth, causing irreversible expansion of the cell wall due to turgor pressure's force and inducing structural changes that modify its extensibility (Jordan and Dumais, 2010). Although turgor has long been assumed to be a rather passive contributor to cell shaping, recent reports show that, in some cells, differential changes in turgor may have a role in establishing specialized cell form (Martin et al., 2001).

Several articles have shown the comparison between VGGs and ResNET for image classification (Wieslander et al., 2017; Reddy and Juliet, 2019; Ikechukwu et al., 2021; Jusman, 2023) and have had similar performances compared with our research. Wieslander et al. (2017) showed that ResNet was shown to be the preferable network, with a higher accuracy and a smaller standard deviation than VGG. Ikechukwu et al. (2021) compared three ANNs (VGG-19 ResNet-50 IykeNet) performed very well, but VGG-19 had higher accuracy, specificity, precision, and recall. Jusman (2023) unveiled that ResNet-101 acquired the greatest results with an average accuracy of 97.70%, precision of 93.19%, recall of 93.25%, specificity of 98.62%, and F-score of 93.11%, demonstrating its superiority over VGG-19 in classifying prostate cell images based on testing data.

One disadvantage of VGG architecture is that it cannot get too deep in layers because it starts to lose the generalization capability, i.e., it starts overfitting (Qian et al., 2020; Pardede et al., 2021; Santos and Papa, 2022). This is because as the ANN gets deeper, gradients from the loss function start to shrink to zero, and the weights are not updated (Zhang et al., 2023). This is known as the notorious problem of vanishing/exploding gradients (Bengio et al., 1994; Glorot and Bengio, 2010; Basodi et al., 2020). ResNet solved this problem by using skip connections (Jakubec et al., 2021; Santos-Bustos et al., 2022).

## 5 Conclusion

*Tradescantia* revealed sensibility after exposure to air pollution proxy PM and diesel-contaminated soil. Pink cell appearance presented a higher correlation with PM_10_ when compared with PM_2.5_. The experiments described here provided images for constructing the single-cell Trad-204 dataset, which can be used as a resource for testing and validating automated image-analysis algorithms. It is the first dataset containing single cell images from *Tradescantia* clone 4430, a biomarker and biomonitor for environmental changes and stressors.

Among the graphs used for color classification, ResNET-34 had 100% accuracy in classifying a subset of 106 images from the Trad-204 dataset and also achieved high validation accuracy with the least number of epochs. Other ANN architectures such as TinyVGG and VGG16 demonstrated good performances (accuracy between 77% and 98%) while the baseline model had the worst performance.

For further studies, other ANN architectures can be explored while the *Tradescantia* stamen hair cell dataset can be expanded with images from new experiments. Data augmentation and different normalization strategies can be adopted to improve image recognition and generalization. Another step in the research is to use algorithms for cell segmentation, such as Contour Proposal Networks (CPNs), to crop the cells from stamen hair images and then apply neural networks to classify cell colors. Another suggestion is to create labels for cell shapes and test recognition of shape patterns using artificial neural networks. There is evidence showing that environmental stress factors can cause changes in biological cell shape into giant, dwarf, bent, benched, and stunted cells (Caldwell et al., 1974; Cosgrove, 1997; Mykytczuk et al., 2007; Cook et al., 2008).

The methods presented here can be adapted in other studies that require the identification and counting of plant or animal cells. Environmental biomonitoring works in conjunction with the physical or chemical monitoring of environmental stressors, whether they are present in the air, water, or soil. This research helps to get quicker and more accurate results with bioindicators so that we can act more effectively through actions in reducing pollutant sources. Future and strengthening of biomonitoring in public policies and regulations depends on continuous development, standardizing the techniques used for each bioindicator. This will improve the cost-benefit ratio for its application as an environmental decision-making tool, demonstrating that biomonitoring is a crucial tool for highlighting environmental changes caused by anthropogenic actions.

## Data availability statement

The datasets generated and analyzed for this study can be found in the GitHub repository: https://github.com/emiliomercuri/Trad-204.

## Author contributions

LR: Conceptualization, Methodology, Writing – review & editing. BG: Methodology, Writing – review & editing. EM: Conceptualization, Methodology, Project administration, Writing – original draft. SN: Conceptualization, Methodology, Project administration, Writing – review & editing.
